# Synergistic Activity of Imipenem in Combination with Ceftazidime/Avibactam or Avibactam against Non-MBL-Producing Extensively Drug-Resistant Pseudomonas aeruginosa

**DOI:** 10.1128/spectrum.02740-21

**Published:** 2022-03-22

**Authors:** Yulin Zhang, Jiankang Zhao, Jiajing Han, Yanyan Fan, Zhujia Xiong, Xiaohui Zou, Binbin Li, Xinmeng Liu, Ziyao Li, Binghuai Lu, Bin Cao

**Affiliations:** a Department of Pulmonary and Critical Care Medicine, Laboratory of Clinical Microbiology and Infectious Diseases, Center for Respiratory Diseases, National Clinical Research Center of Respiratory Diseases, China-Japan Friendship Hospital, Beijing, China; b Institute of Respiratory Medicine, Chinese Academy of Medical Sciences, Peking Union Medical College, Beijing, China; c Clinical Center for Pulmonary Infections, Capital Medical University, Beijing, China; d Tsinghua University-Peking University Joint Center for Life Sciences, Beijing, China; Johns Hopkins Hospital

**Keywords:** ceftazidime-avibactam, extensively drug-resistant *Pseudomonas aeruginosa*, imipenem, synergistic antibacterial activity, antibiotic resistance

## Abstract

Extensively drug-resistant Pseudomonas aeruginosa (XDRPA) infection is a significant public health threat due to a lack of effective therapeutic options. New β-lactam-β-lactamase inhibitor combinations, including ceftazidime-avibactam (CZA), have shown a high resistance rate to XDRPA. This study was therefore conducted to describe the underlying genomic mechanism of resistance for CZA nonsusceptible XDRPA strains that are non-metallo-β-lactamase (MBL) producers as well as to examine synergism of CZA and other antipseudomonal agents. Furthermore, the synergistic antibacterial activity of the most effective antimicrobial combination against non-MBL-producing XDRPA was evaluated through *in vitro* experiments. The resistance profiles of 15 CZA-resistant XDRPA strains isolated from clinical specimens in China-Japan Friendship Hospital between January 2017 to December 2020 were obtained by whole-genome sequencing (WGS) analysis. MBL genes *bla*_IMP-1_ and *bla*_IMP-45_ were found in 2 isolates (2/15, 13.3%); the other underlying CZA-resistance mechanisms involved the decreased OprD porin (13/13), *bla*_AmpC_ overexpression (8/13) or mutation (13/13), and upregulated efflux pumps (13/13). CZA-imipenem (CZA-IPM) combination was identified to be the most effective against non-MBL-producing XDRPA according to the results of WGS analysis and combined antimicrobial susceptibility tests, with an approximately 16.62-fold reduction in MICs compared to CZA alone. Furthermore, the results of checkerboard analysis and growth curve displayed the synergistic antimicrobial activity of CZA and IPM against non-MBL-producing XDRPA. Electron microscopy also revealed that CZA-IPM combination might lead to more cellular structural alterations than CZA or IPM alone. This study suggested that the CZA-IPM combination has potential for non-MBL-producing XDRPA with *bla*_AmpC_ overexpression or mutation, decreased OprD porin, and upregulated efflux pumps.

**IMPORTANCE** Handling the infections by extensively drug-resistant Pseudomonas aeruginosa (XDRPA) strains is challenging due to their complicated antibiotic resistance mechanisms in immunosuppressed patients with pulmonary diseases (e.g., cystic fibrosis, chronic obstructive pulmonary disease, and lung transplant), ventilator-associated pneumonia, and bloodstream infections. The current study suggested the potentiality of the ceftazidime-avibactam-imipenem combination against XDRPA with *bla*_AmpC_ overexpression or mutation, decreased OprD porin, and/or upregulated efflux pumps. Our findings indicate the necessity of combined drug sensitivity tests against XDRPA and also lay a foundation for the development of prevention, control, and treatment strategies in XDRPA infections.

## INTRODUCTION

Pseudomonas aeruginosa is a Gram-negative aerobic bacillus responsible for opportunistic infections in humans. The high morbidity and mortality associated with the organism were noted in immunosuppressed patients with pulmonary diseases (e.g., cystic fibrosis, chronic obstructive pulmonary disease), ventilator-associated pneumonia (VAP), and disseminated infections (1–3). Multidrug-resistant P. aeruginosa is increasingly observed worldwide (4). Handling the infections by extensively drug-resistant P. aeruginosa (XDRPA) is challenging due to their complicated intrinsic and acquired antibiotic resistance mechanisms (4–6).

In the “Bad Bugs, No Drugs” era, there are novel antibiotic agents available, but the treatment option for XDRPA is still limited. P. aeruginosa is intrinsically resistant to tigecycline (7, 8). Colistin remains one of the leading effective agents but is limited with toxicities and agreed dosing regimen for XDRPA (9). Notably, ceftazidime-avibactam (CZA) has been approved for complicated intraabdominal infections, complicated urinary tract infections, and VAP caused by multidrug-resistant Gram-negative bacteria (10, 11). Avibactam maintains the potential efficacy against the class A, C, and partial D β-lactamases but not for class B β-lactamases (11). Results from the China Antimicrobial Surveillance Network (CHINET) revealed that more P. aeruginosa isolates were susceptible to CZA than to ceftazidime (CAZ) (86.5% versus 71.8%) (12). In particular, 65.7% of carbapenem-resistant P. aeruginosa isolates were susceptible to CZA, suggesting the additional role of avibactam against P. aeruginosa (12). However, some XDRPA isolates showed a high resistance rate to CZA (50.9%) (13). There is an urgent need to develop an effective antimicrobial combination treatment strategy for these organisms.

The combination of aztreonam and CZA has been confirmed to achieve a synergistic antibacterial activity against various drug-resistant P. aeruginosa strains with metallo-β-lactamase (MBL) genes (14–16). Therefore, resistance mechanisms of 13 non-MBL-producing CZA-resistant XDRPA strains were genetically elucidated in this study. Subsequently, the synergisms of CZA and other antipseudomonal agents (aztreonam, amikacin, piperacillin-tazobactam, imipenem, and meropenem) against these XDRPA strains were compared by combined drug sensitivity tests. Furthermore, the synergistic antibacterial activity of the most effective antimicrobial combination was evaluated through *in vitro* experiments.

## RESULTS

### Resistance mechanisms among XDRPA isolates.

A total of 15 CZA-resistant XDRPA strains were involved in the study, 2 of which were MBL producers. The demographic, clinical, and strain characteristics of the 15 XDRPA-infected patients are described in Table 1. Overall, all patients had 1 or more coexisting underlying diseases (e.g., cardiac disease, diabetes, hypertension, and so on; Table 1). Four patients died during hospitalization. MLST analysis revealed that 15 XDRPA strains belonged to 5 sequence types (STs; ST270, ST773, ST181, ST1182, and ST3405) (Table 1). Antimicrobial susceptibility test (AST) results of the several major antimicrobials (CZA, MEM, IPM, AK, ATM, TZP, and colistin) against all XDRPA isolates are also listed in Table 1.

**TABLE 1:** Demographic, clinical, and strain characteristics of the patients infected by 15 extensively drug-resistant P. aeruginosa isolates

No	Age (y)/sex	Underlying diseases	Present disease	Outcomes	ST	Presence of resistance mechanism:							MIC (μg/mL)^*a*^ of:						
						*OprD* mutation	*bla*_AmpC_ T105A mutation	*bla*_AmpC_ with Ω-loop substitution (E247K)	*bla*_AmpC_ overexpression	*bla*_PER-1_	Efflux pumps overexpression	MBL	CZA	MEM	IPM	AK	ATM	TZP	COS
PA04	78/F	Renal insufficiency, diabetes, cardiac disease, hypertension	Severe pneumonia	Death	270	+	+	−^*c*^	−	−	+	−	32(R)	4(I)	8(R)	8(S)	16(I)	128(R)	≤2(I)
PA05	55/M	Chronic pulmonary heart disease, interstitial lung disease	Lung transplant status	Cured	181	+	+	−	−	−	−	+^*b*^	1024(R)	256(R)	128(R)	>128(R)	>32(R)	128(R)	≤2(I)
PA06	60/M	Bronchiolitis, hypertension, diabetes, hepatitis	Lung transplant status	Cured	270	+	+	−	15.89×	−	+	−	32(R)	8(R)	8(R)	32(I)	32(R)	256(R)	≤2(I)
PA11	31/M	Bronchiectasis, renal injury	Lung transplant status	Cured	1182	+	+	+	−	−	+	−	128(R)	16(R)	8(R)	>128(R)	>32(R)	64(I)	≤2(I)
PA12	69/M	Renal insufficiency, cardiac disease	Severe pneumonia	Cured	270	+	+	−	85.24×	−	+	−	32(R)	8(R)	8(R)	8(S)	32(R)	128(R)	≤2(I)
PA13	66/M	Lymphoma, intestinal obstruction	Severe pneumonia	Death	773	+	+	−	−	+	+	−	32(R)	8(R)	16(R)	>128(R)	>32(R)	64(I)	≤2(I)
PA17	61/M	Hypertension, cardiac disease, diabetes	Pneumonia	Cured	270	+	+	−	51.20×	−	+	−	32(R)	8(R)	8(R)	8(S)	16(I)	128(R)	≤2(I)
PA18	85/M	Renal failure, hypertension, cardiac disease, diabetes	Severe pneumonia	Death	270	+	+	−	17.58×	−	+	−	32(R)	8(R)	8(R)	16(S)	>32(R)	32(I)	≤2(I)
PA19	50/M	Renal failure, hypertension, cardiac disease	Severe pneumonia	Cured	270	+	+	−	11.83×	−	+	−	32(R)	8(R)	8(R)	8(S)	16(I)	128(R)	≤2(I)
PA22	51/F	Bronchial asthma	Pneumonia	Cured	270	+	+	−	14.74×	−	+	−	32(R)	8(R)	16(R)	16(S)	>32(R)	32(I)	≤2(I)
PA24	87/M	Hypertension, diabetes, cardia-cerebrovascular disease	Severe pneumonia	Cured	270	+	+	−	235.40×	−	+	−	32(R)	8(R)	8(R)	16(S)	>32(R)	128(R)	≤2(I)
PA25	63/F	Renal insufficiency, diabetes, cardiac disease	Lung transplant status	Death	270	+	+	−	−	−	+	−	32(R)	8(R)	8(R)	16(S)	>32(R)	128(R)	≤2(I)
PA27	78/M	Renal insufficiency, hypertension, cardiac disease	Aspiration pneumonitis	Cured	270	+	+	−	−	−	+	−	32(R)	16(R)	16(R)	32(I)	16(I)	32(I)	≤2(I)
PA29	19/M	Renal insufficiency	Severe pneumonia	Cured	270	+	+	−	−	−	+	−	32(R)	16(R)	16(R)	128(R)	>32(R)	128(R)	≤2(I)
PA38	84/F	Hypertension, diabetes, cardia-cerebrovascular disease	Severe pneumonia	Cured	3405	+	+	−	62.92×	−	+	+^*b*^	2048(R)	>256(R)	256(R)	32(I)	>32(R)	256(R)	≤2(I)

a R, resistant; S, susceptible; I, intermediate.

b PA05 and PA38 strains are positive for MBL.

c −, negative for corresponding resistance mechanism.

**FIG 1:**
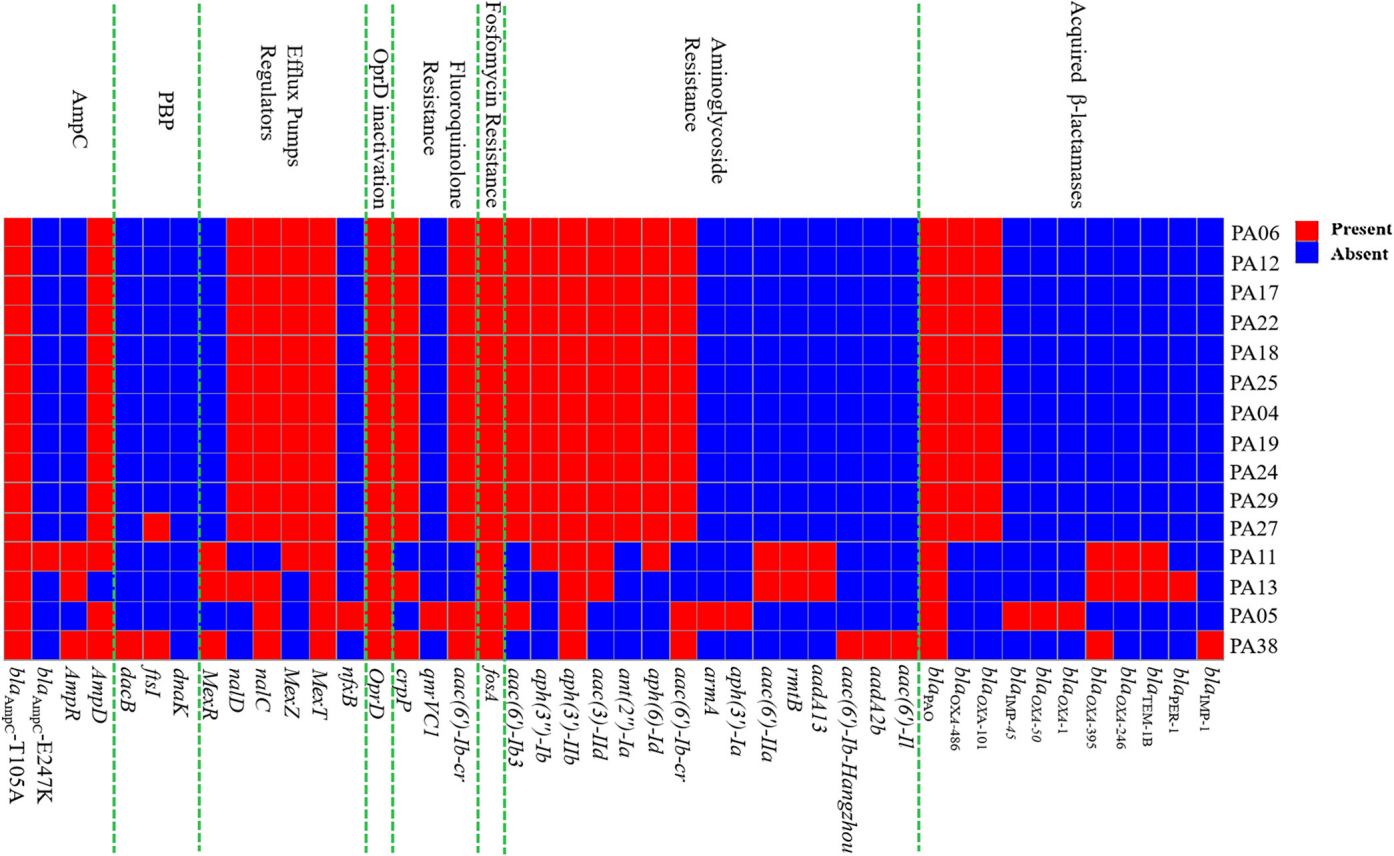
Main antibiotic resistance genes detected among the 15 extensively drug-resistant P. aeruginosa (XDRPA) isolates.

As shown in Fig. 1, genes resistant to quinolones, fosfomycin, aminoglycosides, and β-lactams among these XDRPA isolates were presented. β-Lactamase genes intrinsic to this species, including *bla*_PAO_, *bla*_OXA-50_, and *bla*_OXA-50_-like (*bla*_OXA-486_ and *bla*_OXA-395_), were detected in all isolates. MBL genes *bla*_IMP-1_ and *bla*_IMP-45_ were found in 2 isolates (2/15, 13.3%). Other β-lactamase genes identified included *bla*_TEM-1B_ (2 isolates), *bla*_PER-1_ (1), *bla*_OXA-1_ (1), *bla*_OXA-101_ (11), and *bla*_OXA-246_ (2). In addition, the T105A substitution in *bla*_AmpC_ gene that might hydrolyze IPM and the mutations of porin *OprD* were identified in all XDRPA strains. Moreover, the mutations of *ampR* and *ampD* genes regulating *bla*_AmpC_ expression were also identified (Fig. 1), and 8/15 (53.3%) displayed *bla*_AmpC_ overexpression (Table 1). Notably, one strain, PA11, displayed a unique alteration (E247K) in the *bla*_AmpC_ Ω-loop, associated with the resistance to CZA. In addition, the frequent mutations in the regulatory components of efflux pump (*mexR*, *mexZ*, *mexT*, *nalC*, and *nalD* genes) were detected in these strains (Fig. 1). One or more kinds of efflux pumps were found to be overexpressed in these strains (Table 1). Overall, the overexpression of *mexA*, *mexC*, *mexE*, and *mexX* was observed in 2 (13.3%), 1 (6.7%), 4 (26.7%), and 11 (73.3%) strains.

### Efficacy of different antimicrobial combinations.

**FIG 2:**
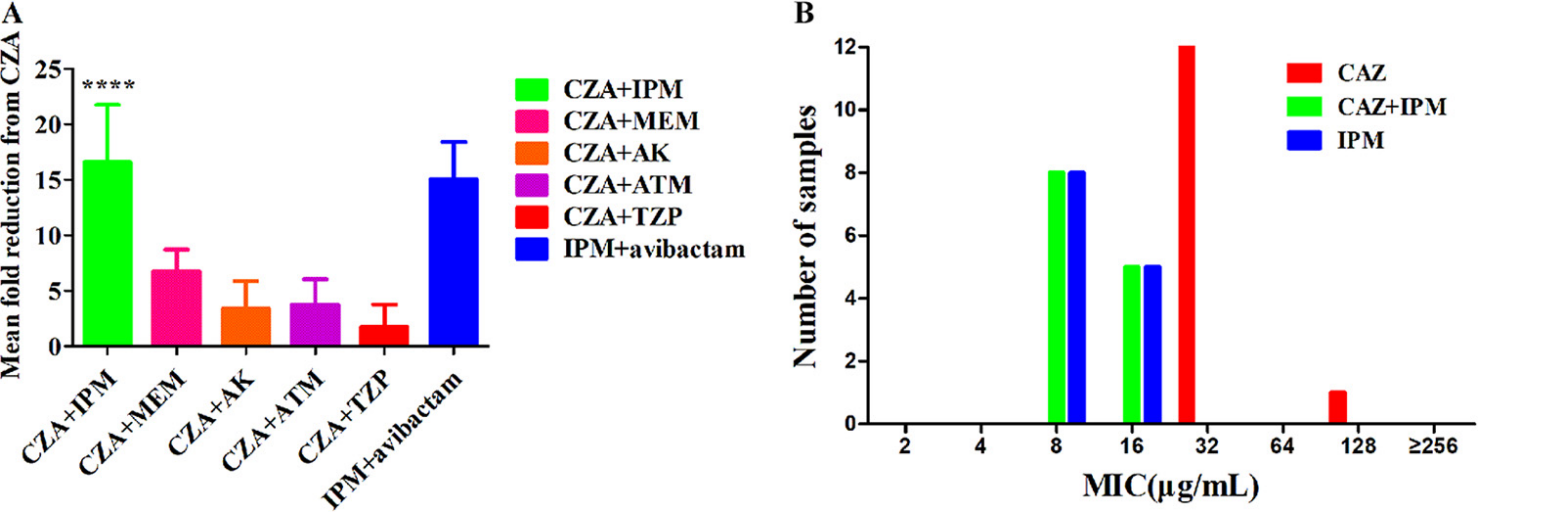
MIC reductions of CZA in combination with other antimicrobials (A) and MIC values of CAZ and IMP alone and in combination (B) against 13 extensively drug-resistant P. aeruginosa (XDRPA) isolates without metallo-β-lactamase. ATM, aztreonam; IPM, imipenem; MEM, meropenem; AK, amikacin; TZP, piperacillin-tazobactam; CAZ, ceftazidime. ****, CZA-IPM combination exhibited the greatest reduction of the MIC values (with the mean reduction of 16.62 ± 5.12 fold) compared to the combination of CZA and other drugs (*P < * 0.05).

Given that β-lactam resistance conferred by MBL in P. aeruginosa could be overcome by CZA and ATM combination, even though avibactam could not inhibit class B β-lactamases (17), 13 non-MBL-producing XDRPA isolates in this study were used for exploring the most effective therapeutic strategy. As described in Fig. 2A and Table 2, CZA-IPM combination exhibited a 16.62 ± 5.12-fold reduction of the CZA-MIC values compared to the combination of CZA and other drugs (*P < *0.05). However, the addition of ceftazidime to IPM could not reduce the IPM-MIC values of all isolates (Fig. 2B), whereas the addition of avibactam to IPM reduced the MIC values of 11 isolates (Fig. 2A and Table 2). The MIC values of PA11 (*bla*_AmpC_ with Ω-loop substitution E247K) and PA13 (*bla*_PER-1_ encoding extended-spectrum β-lactamase [ESBL]) could not be decreased by the IPM and avibactam combination (Table 2). As a result, the antimicrobial activity of CZA-IPM combination against 13 XDRPA isolates was further assessed with the methods of checkerboard analysis and growth curve.

**TABLE 2:** MIC of CZA alone and in combination with other antimicrobials against 13 extensively drug-resistant P. aeruginosa isolates

ID	MIC (μg/mL) of:											
	CZA	CZA+IPM	Avibactam+IPM	IPM	CZA+MEM	MEM	CZA+AK	AK	CZA+TZP	TZP	CZA+ATM	ATM
PA04	32	2	2	8	4	4	8	8	32	128	8	16
PA06	32	2	2	8	4	8	32	32	32	256	8	32
PA11	128	4	8	8	16	16	>128	>128	16	64	32	>32
PA12	32	2	2	8	4	8	8	8	32	128	8	32
PA13	32	4	8	16	8	8	>128	>128	16	64	16	>32
PA17	32	2	2	8	4	8	8	8	16	128	4	16
PA18	32	2	2	8	4	8	4	16	16	32	4	>32
PA19	32	2	2	8	4	8	8	8	32	128	8	16
PA22	32	2	2	16	8	8	4	16	16	32	8	>32
PA24	32	2	2	8	4	8	8	16	64	128	>32	>32
PA25	32	2	2	8	8	8	16	16	128	128	>32	>32
PA27	32	2	2	16	4	16	16	32	16	32	8	16
PA29	32	2	2	16	8	16	16	128	128	128	>32	>32

### Checkerboard analysis of CZA-IPM combination.

**FIG 3:**
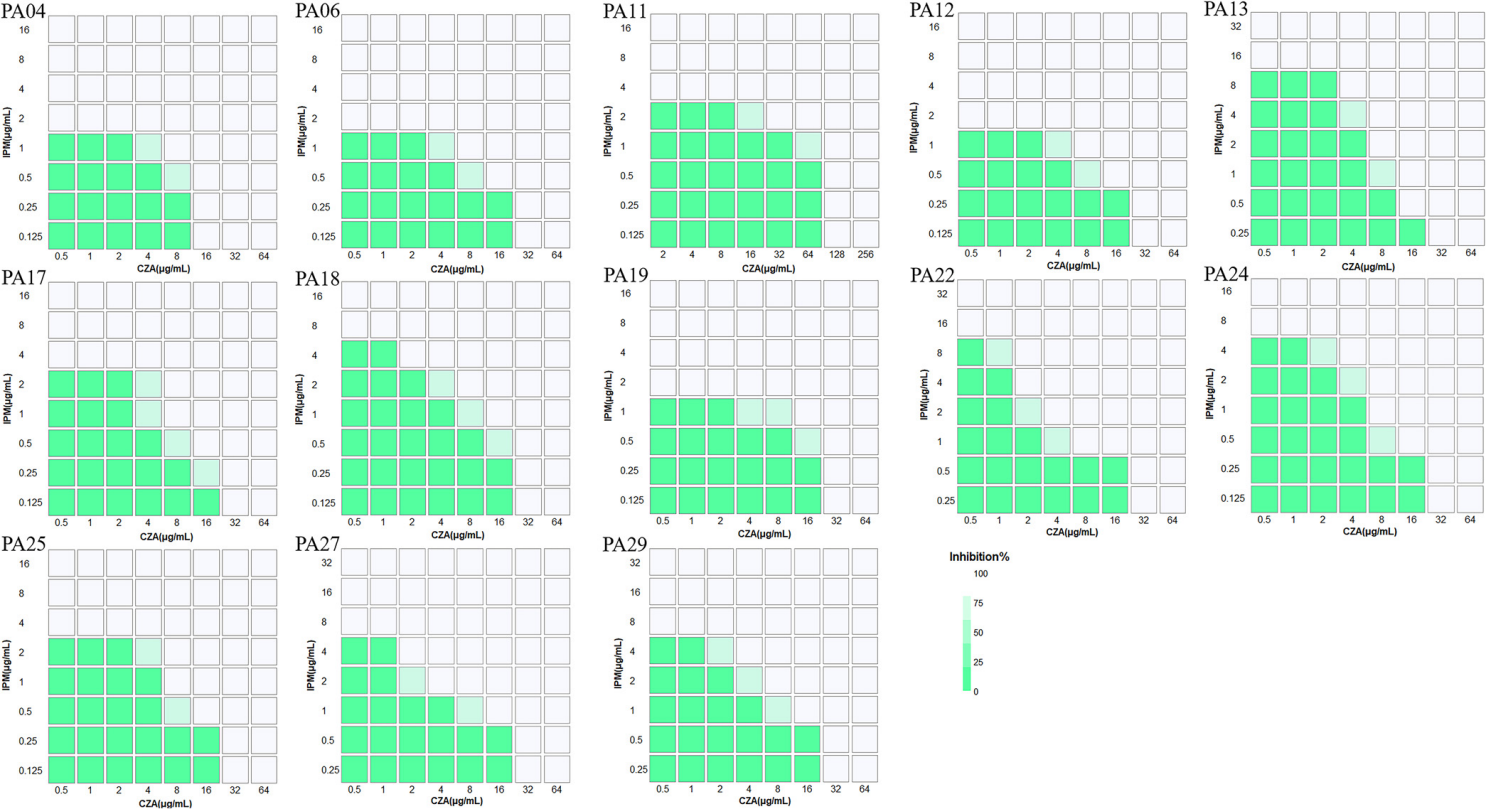
Heat plots of microdilution checkerboard assays for ceftazidime-avibactam (CZA) and imipenem (IPM) combination against 13 extensively drug-resistant P. aeruginosa (XDRPA) strains without metallo-β-lactamase.

Checkerboard analysis experiments of the above 13 XDRPA isolates were performed to evaluate whether the CZA-IPM combination was synergistic or not. As described in Fig. 3, the results revealed that the CZA-IPM combination possessed a ≤0.5 fractional inhibitory concentration index (FICI) for each strain, showing the synergistic antibacterial activity of CZA and IPM against these XDRPA strains.

### Growth curve analysis.

**FIG 4:**
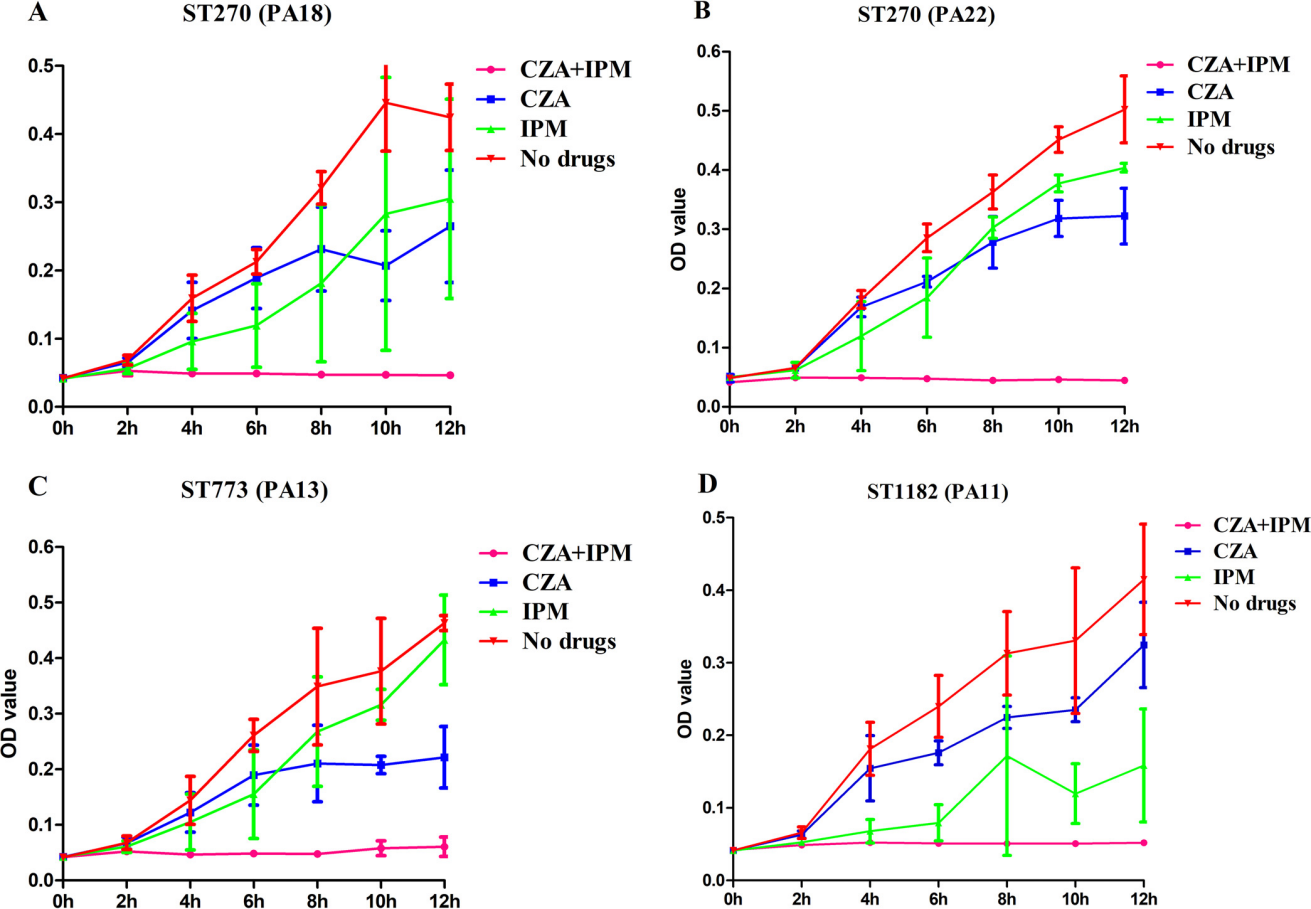
Analysis of growth curve. (A to D) The growth of 4 extensively drug-resistant P. aeruginosa strains (PA18, PA22, PA13, and PA11) of different STs (ST270, ST270, ST773, and ST1182) in cation-adjusted Mueller-Hinton broth (CAMHB) with 0.5 MIC of ceftazidime-avibactam (64/4, 16/4, 16/4, and 16/4 μg/mL), 0.5 MIC of imipenem (4, 8, 4, and 8 μg/mL), or 0.5 MIC of ceftazidime-avibactam (64/4, 16/4, 16/4, and 16/4 μg/mL) and 0.5 MIC of imipenem (4, 8, 4, and 8 μg/mL) combination, respectively, shaking at 37°C for 12 h. OD_600_ values were measured at 2, 4, 6, 8, 10, and 12 h after shaking, respectively.

As depicted in Fig. 4, compared to the control group, CZA and IPM combination inhibited the growth of 4 XDRPA strains of 3 different STs for up to 12 h (*P < *0.05). In contrast, treatment with CZA or IPM alone could not entirely inhibit their growth within 12 h.

### Scanning electron microscopy analysis of PA11 and PA22.

**FIG 5:**
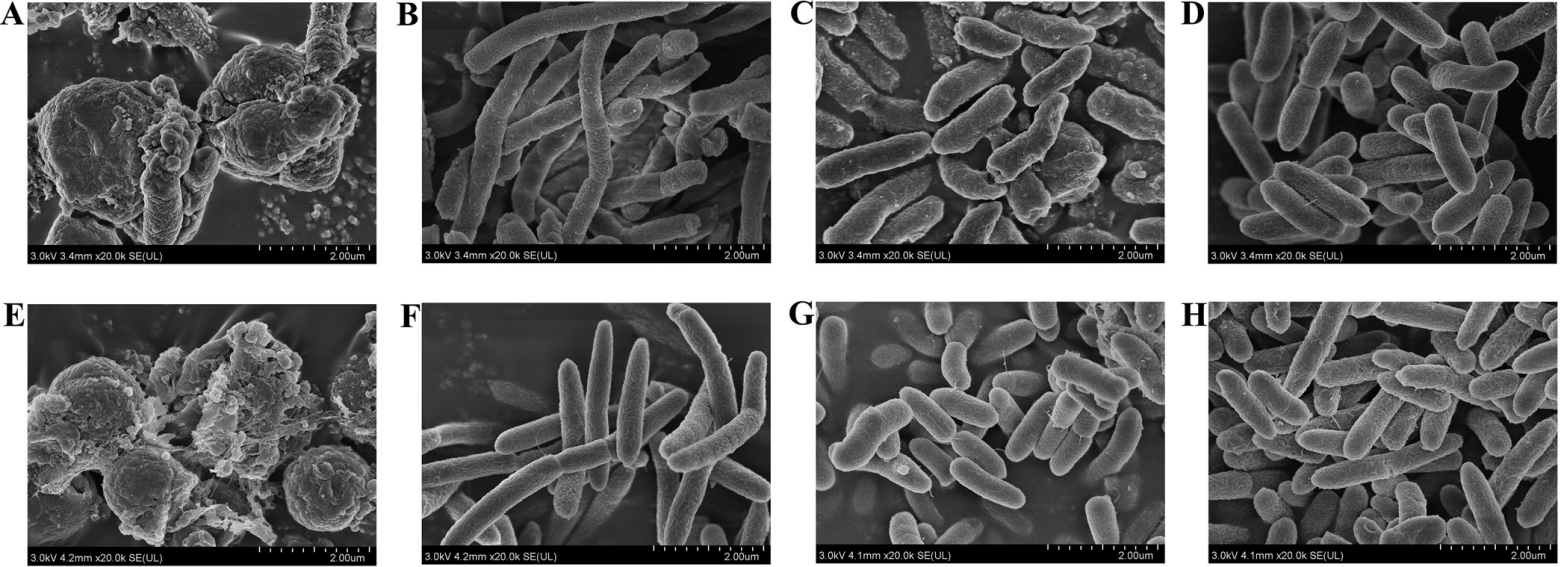
SEM images of 2 ceftazidime-avibactam-resistant extensively drug-resistant P. aeruginosa strains (PA11: ST1182 and PA22: ST270) after treatment with ceftazidime-avibactam, imipenem, or ceftazidime-avibactam-imipenem and no drugs for 4 h. (A to D) SEM images of PA11 strain treated by ceftazidime-avibactam (32 μg/mL and 4 μg/mL), imipenem (2 μg/mL), ceftazidime-avibactam-imipenem (32 μg/mL, 4 μg/mL, 2 μg/mL), and no drugs. (E to H) SEM images of PA22 strain treated by ceftazidime-avibactam (8 μg/mL and 4 μg/mL), imipenem (4 μg/mL), ceftazidime-avibactam-imipenem (8 μg/mL, 4 μg/mL, 4 μg/mL), and no drugs.

CZA or IPM monotherapy had no effect on the integrity of the cellular surface with the minor morphological change (Fig. 5B, C, F, and G), in contrast with that of the control group (Fig. 5D and H). CZA-IPM combination, nevertheless, induced cell shrinkage and cell surface bulging and increased particles, large-scale membrane disruptions, and bacterial cell lysis (Fig. 5A and E).

### CZA and IPM tolerance in mice.

**FIG 6:**
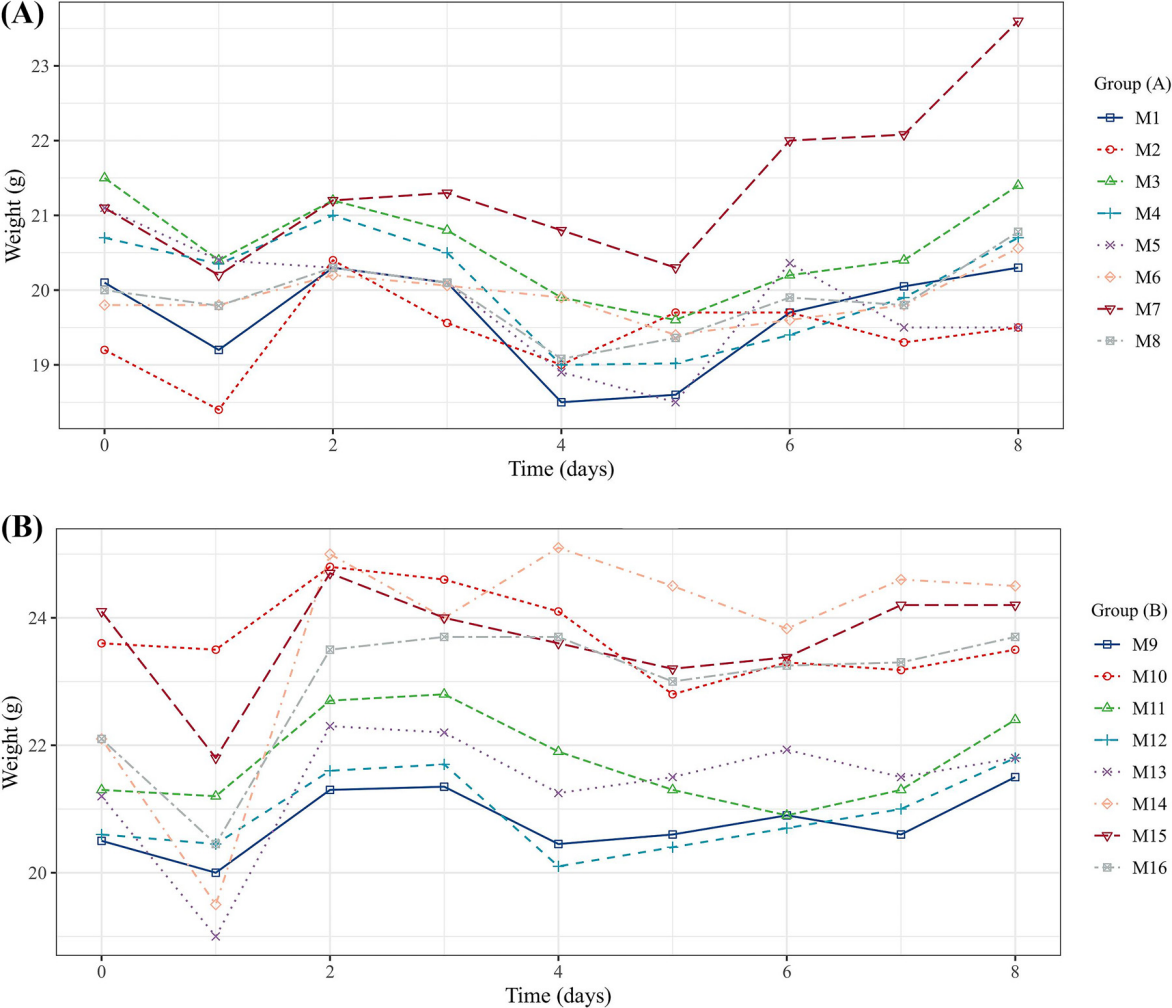
Bodyweight changes in the neutropenic mice receiving intraperitoneal injection by CZA and IPM combination (A) or sterile saline solution (B), respectively, three doses per day for 3 days.

Compared with the control group administered with sterile saline solution, the tested neutropenic mice receiving intraperitoneal injection by CZA and IPM with three doses each day for 3 days had no adverse reactions, including the body weight loss and mental abnormalities (Fig. 6).

## DISCUSSION

In recent years, the increasing prevalence of XDRPA infections has posed a severe clinical challenge worldwide, particularly in immunocompromised patients (18–21). CZA exhibited good activity against P. aeruginosa; however, CZA-resistant isolates have been reported, especially in XDRPA (10, 22, 23). A total of 15 XDRPA isolates were collected to be resistant to CZA in this study, 4 of which were from the lung transplantation patients, and 1 isolate (PA13) belonged to the high-risk clone ST773 (24). The effective antimicrobial combinations with synergistic activity fighting diverse mechanisms are the potential choice. Recently, aztreonam and CZA in combination has been confirmed to be a viable treatment option against MBL-producing P. aeruginosa (16, 18, 25). As a consequence, this study described underlying genomic CZA resistance mechanism for 13 non-MBL-producing XDRPA strains and compared the synergism of CZA and other antipseudomonal agents (aztreonam, amikacin, piperacillin-tazobactam, imipenem, and meropenem), respectively. On this basis, the synergistic antibacterial activity of the most effective antimicrobial combination against XDRPA was further assessed through *in vitro* experiments.

According to the genomic resistance profile, multiple resistance mechanisms, including the hyperexpression of efflux pumps, *OprD* mutations, and the existence of genes resistant to quinolones, fosfomycin, aminoglycosides, and β-lactams, could concomitantly contribute to the antimicrobial resistance in these XDRPA isolates (26). Among β-lactams, OXA-1 could efficiently hydrolyze only oxacillin. OXA-50 and OXA-50-like have no hydrolysis activity to CAZ. OXA-101 and OXA-246, as OXA-10 ESBL derivatives, have no carbapenemase activity and could not confer reduced susceptibility to CZA (27–29). Also, TEM-1B ESBL could be inhibited by IPM and CZA (30). Whereas PER-1 ESBL has been confirmed to be a possible source of CZA resistance, this enzyme could be inhibited by IPM (30). The AmpC enzyme with T105A substitution hydrolyzing IPM could be inhibited by CZA (31). AmpC derepression could be associated with the IPM and CAZ resistance. Overall, the AmpC enzyme with T105A substitution, *bla*_AmpC_ overexpression, and *OprD* mutation could be the underlying reason for IPM resistance in these XDRPA isolates (31, 32). AmpC derepression, the existence of PER-1 ESBL, hyperexpression of efflux pumps, and *OprD* mutations could lead to CAZ or CZA resistance (30, 33–36). In addition, CZA resistance also occurred due to the failure of avibactam to inhibit AmpC enzyme with *bla*_AmpC_ Ω-loop substitutions (E247K) in PA11 strain (37).

**FIG 7:**
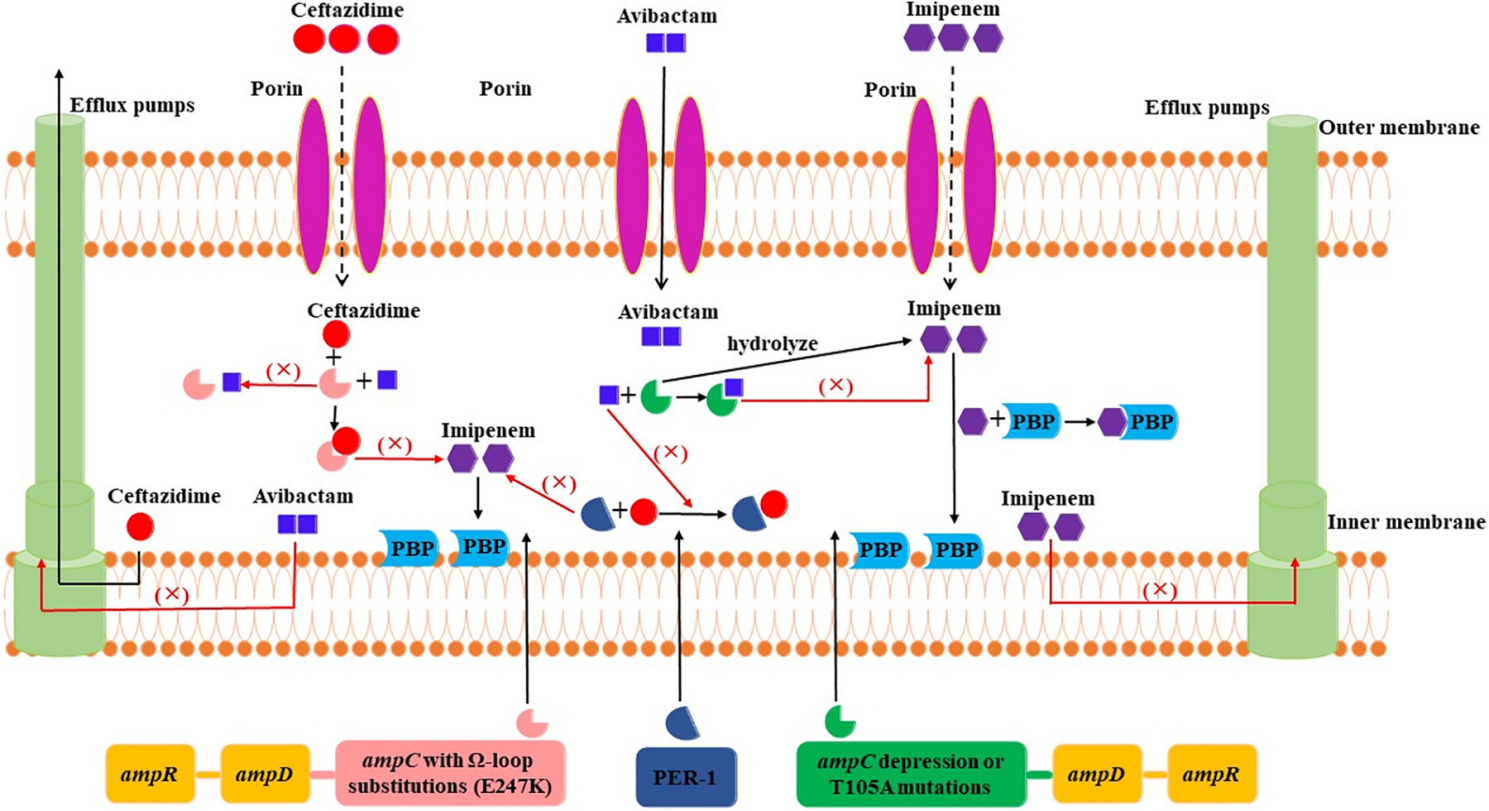
Mechanisms of ceftazidime-avibactam-imipenem combination against extensively drug-resistant P. aeruginosa strains, explained as follows: avibactam inhibits the IPM-hydrolyzing AmpC enzymes (including the *bla*_AmpC_ T105A mutant), thus restoring the antimicrobial activity of IPM. Moreover, avibactam activity is not be affected by the decreased OprD porin; IPM is unaffected by the upregulated efflux pumps given that IPM is a poor substrate for efflux pumps. Furthermore, IPM could not be hydrolyzed by ESBL PER-1 in the PA13 strain. Limited IPM crosses the outer membrane of P. aeruginosa strains with *OprD* mutations, whereas intracellular levels of CAZ decrease due to the upregulation of efflux systems and decreased OprD porin. Therefore, the avibactam-IPM combination is superior to CZA against isolates with efflux hyperexpression and decreased OprD porin; the hydrolysis of CAZ is largely enhanced by AmpC with Ω-loop substitution (E247K) in PA11 strain, which perhaps reduces carbapenems hydrolysis; therefore, IPM susceptibility could be restored in this strain.

Taken together, we speculated that CZA-IPM combination could be effective against XDRPA in the present study based on the following facts (Fig. 7). (i) Avibactam inhibits the IPM-hydrolyzing AmpC enzymes (including the *bla*_AmpC_ T105A mutant) (38) and thus restores the antimicrobial activity of IPM. In addition, avibactam activity is not be affected by the decreased OprD porin (39). (ii) IPM is not affected by the upregulated efflux pumps given that IPM is a poor substrate for efflux pumps. Furthermore, IPM could not be hydrolyzed by ESBL PER-1 in the PA13 strain. Limited IPM crosses the outer membrane of P. aeruginosa strains with *OprD* mutations, whereas intracellular levels of CAZ decrease due to the upregulation of efflux systems and decreased OprD porin (40). Therefore, the avibactam-IPM combination is superior to CZA against isolates with efflux hyperexpression and decreased OprD porin. (iii) The hydrolysis of CAZ is enhanced largely by AmpC with Ω-loop substitution (E247K) in PA11 strain, which perhaps reduces carbapenems hydrolysis; therefore, IPM susceptibility could be restored in this strain. Similar findings have been described in previous studies, which demonstrated that the CZA-IPM combination could be a useful therapeutic option for KPC-producing Klebsiella pneumoniae (D179Y mutation in *bla*_KPC_ gene) infections compared with the combinations of CZA and other antimicrobial drugs (gentamicin, ciprofloxacin, ertapenem, and tigecycline) (41). Overall, the CZA-IPM combination could be an attractive therapy option for XDRPA infection with *bla*_AmpC_ overexpression or mutation, decreased OprD porin, upregulated efflux pumps, and the existence of PER-1 ESBL. These multiple resistant mechanisms were also prevalent in other XDRPA strains worldwide (26, 42).

This hypothesis proved to be correct. CZA-IPM displayed higher antibacterial activity against CZA-resistant XDRPA than other CZA-containing combinations (Fig. 2). Moreover, the synergistic antibacterial effect of IPM and CZA has also been confirmed in our *in vitro* study. Similar findings have been described in previous studies, which reported synergistic effects between CZA and IPM combination in KPC-producing K. pneumoniae isolates (41). Another study showed that the hydrolysis of IPM could be inhibited by avibactam in *bla*_AmpC_-induced P. aeruginosa strains, which might be the underlying mechanism of the synergistic activity between CZA and IPM combinations (43). Consistent with this view, avibactam rather than ceftazidime could restore the antibacterial activity of IPM in this current study by inhibiting AmpC (Fig. 2 and 7), hinting that avibactam and IPM in combination could be an ideal therapeutic strategy for XDRPA as IPM/relebactam, which is proved to play a vital role in therapy against XDRPA (38). Given that the activity of avibactam is higher than that of relebactam (44), IPM-CZA combination might, therefore, be the appropriate treatment option for XDRPA infections. Additionally, a previous study revealed that an additive or synergistic effect in 7 XDRPA strains with different resistance mechanisms was obtained by different combination therapies, including CZA-colistin, CZA-amikacin, and CZA-aztreonam (45). Nevertheless, whether CZA-IPM combination is effective for combining those non-MBL-producing strains needs further research.

Strengths of this study included the CZA-resistant XDRPA isolates with diverse resistance mechanisms, sufficient evidence (the microbroth checkerboard dilution, growth curve, and SEM *in vitro*), and identification of drugs interaction mechanisms supporting the conclusion. The first limitation is that no CZA-resistant strains conferred by *bla*_KPC_ or *bla*_OXA_ Ω-loop mutations were included in this study. Second, the sample size is small. Although small sample size is common in this type of study, the conclusions have been interpreted with caution. Thus, further study on combination therapy is needed to be performed in more CZA-resistant P. aeruginosa strains with these mutants, such as XDRPA strains carrying *bla*_OXA-14_ and *bla*_OXA-681_.

In summary, this study suggested the potentiality of the CZA-IPM combination in the infections by non-MBL-producing XDRPA isolates with *bla*_AmpC_ overexpression or mutation, decreased OprD porin, upregulated efflux pumps, and the existence of PER-1 ESBL. More studies on the mechanisms of enhanced activity between CZA-IPM combination as well as exploring basic and clinical IPM-avibactam combination will be further performed in future.

## MATERIALS AND METHODS

### Ethical approval.

The animal experiments were approved by the Committee of Laboratory Animal Welfare and Ethics, China-Japan Friendship Hospital (ZRYHYY11-20-07-1). This study was performed in strict accordance with the protocol for the review on Laboratory Animal Welfare and Ethics, China-Japan Friendship Hospital.

### Bacterial isolates and critical antimicrobial agents.

Fifteen XDRPA isolates, defined as nonsusceptibility to ≥1 agent in all classes but ≤2 categories including colistin (18), collected from China-Japan Friendship Hospital from January 2017 to December 2020, were used in this study. The compounds of ceftazidime, avibactam, and imipenem were purchased from MedChemExpress (MCE). These drugs were dissolved in sterile solvents to generate a stock solution, and solutions with various concentrations were further prepared in Mueller-Hinton broth. Others (aztreonam, amikacin, meropenem, piperacillin-tazobactam) were from Wenzhou KONT Biology & Technology Co., Ltd.

### Analysis of WGS.

WGS of all enrolled XDRPA isolates was performed on a HiSeq sequencer (Illumina) following the manufacturer’s instructions. FASTQ format files of each sample were independently assembled using *de novo* assembler SPAdes Genome Assembler v3.13.1. National Centre for Biotechnology Information Bacterial Antimicrobial Resistance Reference Gene Database (https://cge.cbs.dtu.dk/services/ResFinder/) was used to search for potential matches applying the criteria of 90% identity and 60% minimum coverage length to obtain the acquired antimicrobial resistance genes. In addition, the sequences of *ampD*, *bla*_AmpC_, *ampR*, *dacB*, *ftsl*, *dnaK*, *mexR*, *nalD*, *nalC*, *mexZ*, *mexT*, *nfxB*, and *OprD* were extracted from the assembled files and aligned with reference strain PAO1 using SnapGene software version 3.2.1 (from Insightful Science; available at https://snapgene.com). We subsequently further identified the sequence types (STs) and allelic numbers by querying the online multilocus sequence typing (MLST) database (https://pubmlst.org/paeruginosa/).

### Gene expression analysis of *bla*_AmpC_, *mexA*, *mexC*, *mexE*, and *mexX*.

Total RNA was extracted from the late-log phase of bacterial cultures in Luria Bertani broth using RNeasy protect bacteria minikit (Qiagen, Inc.). The RNA concentrations of all samples were obtained using the NanoDrop spectrophotometer. DNase I treatment was performed to remove the residual DNA following the manufacturer’s protocol instructions (Thermo Fisher Scientific, Inc.). RevertAid first-strand cDNA synthesis kit and SYBR green real-time PCR master mixes (Thermo Fisher Scientific, Inc.) were used to evaluate the expression levels of *bla*_AmpC_, *mexA*, *mexC*, *mexE*, and *mexX* genes in all non-MBL-producing XDRPA isolates. PCRs were carried out using ABI QuantStudio5 Q5 real-time PCR detection system. The mRNA levels were considered overexpressed significantly when a 10-fold increase (*bla*_AmpC_) or 5-fold increase (*mexA*, *mexC*, *mexE*, and *mexX*) was observed in comparison with the isolate P. aeruginosa PAO1 (25). The reference gene *rspL* was used as the internal control for PCR signal normalization. Relative quantitative levels were obtained with the 2^−ΔΔCT^ method. For each isolate, three separate RNA samples extracted from three independent cultures were used to measure the average expression levels of the above relative genes.

### AST.

The MIC values of CZA, alone and in combination with aztreonam (ATM; 1 to 128 μg/mL), IPM (0.125 to 256 μg/mL), meropenem (MEM;0.125 to 256 μg/mL), amikacin (AK; 1 to 128 μg/mL), or piperacillin-tazobactam (TZP; 0.125 to 256 μg/mL) against 13 non-MBL-producing XDRPA strains, were determined in duplicate using the broth microdilution according to the Clinical and Laboratory Standards Institute (CLSI) guidelines (46). Antimicrobial susceptibility test (AST) was repeated if the MIC value obtained in duplicate was not in agreement. P. aeruginosa ATCC 27853 was used for routine quality control.

### Checkerboard analysis of combination effects.

The MIC values of the CZA-IPM combination against XDRPA isolates were determined using 96-well microtiter plates. Briefly, the 2-fold serially diluted drugs (from 2 MIC to 0.016 MIC, IPM: 32 to 0.125 μg/mL, CZA: 256/4 to 0.5/4 μg/mL) were mixed in a 96-well plate, respectively. The bacterial suspension was added into cation-adjusted Mueller-Hinton broth (CAMHB) to a final concentration of 5 × 10^5^ CFU/mL. After incubation for 16 to 18 h at 37°C, the MIC results were recorded (47). We evaluated the CZA-IPM combination effects by calculating fractional inhibitory concentration index (FICI): FICI = (MIC of drug CZA in the combination/MIC of drug CZA alone) + (MIC of drug IPM in the combination/MIC of drug IPM alone). FICI of ≤0.5, >0.5 and <4, and ≥4 were categorized as synergistic, noninteractive, and antagonistic effects, respectively (48). This experiment was conducted in triplicate on different days. The representative heat plots of microdilution checkerboard assays for 13 XDRPA strains were plotted using R version 3.4.3 software.

### Growth curve.

For the growth curve, 4 strains were randomly selected for analyzing the synergistic bactericidal effects. The detailed procedure is as follows: each bacterium at 1 × 10^5^ CFU/mL (PA11, PA13, PA18, and PA22) grew in CAMHB with 0.5 MIC of CZA (64/4, 16/4, 16/4, and 16/4 μg/mL), 0.5 MIC of IPM (4, 8, 4, and 8 μg/mL), 0.5 MIC of CZA (64/4, 16/4, 16/4, and 16/4 μg/mL), and 0.5 MIC of IPM (4, 8, 4, and 8 μg/mL) combination, respectively, shaking at 37°C for 12 h. Values of optical density at 600 nm (OD_600_) were measured at 2, 4, 6, 8, 10, and 12 h after shaking, respectively. Meanwhile, the same CAMHB without antibiotics was used as the control group. For each isolate, the growth curve was generated from the mean OD values of three independent experiments.

### Scanning electron microscopy.

The effects of the CZA-IPM combination on the cellular morphology of XDRPA were examined by SEM. Sample preparation: two strains (PA11 with high CZA-MIC value and PA22 with low CZA-MIC value) were grown in CAMHB with 0.25 MIC of CZA (32/4 or 8/4 μg/mL), 0.25 MIC of IPM (2 or 4 μg/mL), 0.25 MIC of CZA (32/4 or 8/4 μg/mL) and 0.25 MIC of IPM (2 or 4 μg/mL) combination, or no drugs (as control), respectively. After 4 h of shaking at 37°C, the above samples were centrifuged at 4,000 × *g* for 10 min twice and the supernatants were discarded. The bacterial pellets were fixed with glutaraldehyde at 4°C overnight. Afterward, the samples were centrifuged at 4,000 × *g* for 5 min again, the fixatives were removed, and the bacterial pellets were resuspended in 1-mL sterile phosphoric acid buffer solution (PBS). SEM was conducted by using a Hitachi SU8020 scanning electron microscope.

### Antimicrobial agents’ tolerance in mice.

The study on CZA and IPM tolerance was conducted in outbred 18- to 22-g female neutropenic ICR mice, which were purchased from Charles River Laboratories. A total of 16 ICR mice were administered 150 mg/kg and 100 mg/kg of cyclophosphamide via intraperitoneal injection, 4 days and 1 day before inoculation, respectively, and divided into experimental and control groups, each group eight mice. Two groups were administered intraperitoneally with CZA and IPM and sterile saline solution, 3 doses each day, for 3 days, respectively. The tolerance in mice was assessed by measuring the body weight and observing the changes in mental state, including delirium, dementia, and coma (49).

### Statistical methods.

All data were processed by SAS 9.1. Two sample independent *t* tests were performed to compare the mean fold reduction of CZA-MIC levels by different antimicrobial drugs as well as the OD values between the two groups in the analysis of growth curve.

### Data availability.

Genome sequences for all involved isolates in this study have been registered under the BioProject number PRJNA763704. The sequence reads of all isolates have been deposited under GenBank accession numbers SAMN21447754 to SAMN21447763, respectively. The original data presented in the study are included in the article. Further inquiries can be directed to the corresponding author.
